# The Interplay of Family History of Clinical Determinants and Cognitive Performance: Insights from the Tata Longitudinal Studies of Ageing in India

**DOI:** 10.1002/alz70857_106261

**Published:** 2025-12-26

**Authors:** Vidhya R, Gauri Mullerpattan, Abhishek Mensegere Lingegodwa, Prathima Arvind, Albert Stezin, Jonas S Sundarakumar, Thomas Gregor Issac

**Affiliations:** ^1^ Centre for Brain Research, Indian Institute of Science, Bangalore, Karnataka, India

## Abstract

**Background:**

A family history of dementia is an important risk indicator for the development of dementia. Additional clinical determinants such as hypertension, diabetes, stroke, cardiac illness, Parkinson's disease, and depression are also associated with cognitive decline. While these conditions are known to impact individual cognition directly, limited research explores the influence of a family history of these clinical conditions on cognitive functioning. Hence, this study investigates whether a familial history of these conditions has an association with cognitive performance.

**Method:**

This cross‐sectional study included 1665 baseline participants aged 45 years and older drawn from the TATA Longitudinal Study of Aging, focusing on non‐demented participants residing in Bangalore. The study integrated detailed family history on clinical conditions (memory issues, stroke, cardiac illness, Parkinson's disease, hypertension, depression, diabetes) and cognitive assessments, (ACE‐III and COGNITO Battery). Descriptive statistics, Chi‐square test, and regression analysis have been used to analyze the data.

**Result:**

Based on preliminary analysis, among 1,665 participants, the gender distribution was nearly balanced, with 50.9% male and 49.1% female participants. 58.1% of participants were under 65 years of age, while 41.1% were aged 65 years or older. The prevalence of family history for various clinical conditions was as follows 17.7% for memory impairment, 60.9% for cardiac illness, 59.4% for stroke, 6.5% for hypertension, and 5.8% for diabetes. A significant difference was observed between the two age groups regarding the family history of memory issues (*p* = 0.012). Also, attention scores differed significantly between individuals with a family history of memory issues (mean = 16.45) and those without (mean = 16.72) (*p* = 0.036).

**Conclusion:**

The analysis revealed a nearly equal gender distribution and a significant proportion of participants under 65 years of age. Family history prevalence was highest for cardiac illness and stroke. Attention scores also varied significantly between individuals with and without a family history of memory issues, aligning with previous research that suggests genetic factors may influence early cognitive performance. Further analysis may help identify early risk markers for cognitive impairment, enabling timely interventions and targeted prevention strategies.

